# Platinum Anniversary: Virus and Lichen Alga Together More than 70 Years

**DOI:** 10.1371/journal.pone.0120768

**Published:** 2015-03-19

**Authors:** Karel Petrzik, Jan Vondrák, Jana Kvíderová, Jaromír Lukavský

**Affiliations:** 1 Department of Plant Virology, Institute of Plant Molecular Biology, Biology Centre of the Czech Academy of Sciences, České Budějovice, Czech Republic; 2 Faculty of Environmental Sciences, Czech University of Life Sciences Prague, Kamýcká 1176, Praha 6, Suchdol, Czech Republic; 3 Institute of Botany, The Czech Academy of Sciences, Třeboň, Czech Republic; Illinois Institute of Technology, UNITED STATES

## Abstract

*Trebouxia aggregata* (Archibald) Gärtner (phylum *Chlorophyta*, family *Trebouxiaceae*), a lichen symbiotic alga, has been identified as host of the well-known herbaceous plant virus *Cauliflower mosaic virus* (CaMV, family *Caulimoviridae*). The alga had been isolated from *Xanthoria parietina* more than 70 years ago and has been maintained in a collection since that time. The CaMV detected in this collection entry has now been completely sequenced. The virus from *T*. *aggregata* is mechanically transmissible to a herbaceous host and induces disease symptoms there. Its genome differs by 173 nt from the closest European CaMV-D/H isolate from cauliflower. No site under positive selection was found on the CaMV genome from *T*. *aggregata*. We therefore assume that the virus’s presence in this alga was not sufficiently long to fix any specific changes in its genome. Apart from this symbiotic alga, CaMV capsid protein sequences were amplified from many other non-symbiotic algae species maintained in a collection (e.g., *Oonephris obesa*, *Elliptochloris *sp., *Microthamnion kuetzingianum*, *Chlorella vulgaris*, *Pseudococcomyxa *sp.). CaMV-free *Chlorella vulgaris* was treated with CaMV to establish virus infection. The virus was still detected there after five passages. The virus infection is morphologically symptomless on *Chlorella *algae and the photosynthesis activity is slightly decreased in comparison to CaMV-free alga culture. This is the first proof as to the natural presence of CaMV in algae and the first demonstration of algae being artificially infected with this virus.

## Introduction

Microalgae (eukaryotic microscopic algae and prokaryotic cyanobacteria) are widely spread in nature, inhabiting all ecosystems from cold, arctic regions to hot springs and arid soils. Free-living microalgae are important CO_2_ consumers, primary biomass producers due to photosynthesis, and producers of various biologically active compounds [1]. In addition to thousands of species of free-living algae, many water (marine) organisms host microalgae as stable hereditary endosymbionts [2]. On land, algal or cyanobacterial colonies are components of lichens’ thalli in association with highly specialized fungi. Nearly 100 species of algae have been reported as photobionts in lichens [3].

Viruses are truly pervasive in aquatic environments and have abundances from 5 x 10^4^ to 1.9 x 10^9^ virus-like particles (VLP) per ml in various water systems [4], [5]. The first isolations of viruses infecting microalgae had been obtained from the marine nanoflagellate *Micromonas pusilla* [6]. Later, *Chlorella* strains isolated from *Hydra viridis* were found to contain VLPs designated *Hydra viridis*-*Chlorella* virus 1 [7]. Moreover, many other marine zoochlorellae have been found to be hosts for double-stranded DNA viruses with very large genomes ranging in size from 170 to 560 kb (review [8], [9]). Most of these viruses lyse algal cells [10] and some of them have been associated with the clearing of algal blooms [11], [12]. Nevertheless, algae-infecting viruses have been identified from less than 1% of known eukaryotic algal species [13]. Furthermore, no virus has heretofore been known for free-living microalgae or for terrestrial symbiotic assemblages like lichens [14]. There also has been no knowledge that viruses of angiosperms are able to infect nonvascular plants (e.g., mosses and algae) either in natural conditions or in the laboratory. No plant virus has been isolated from a nonvascular plant growing in the wild, but Polischuk et al. [15], using ELISA, detected *Tobacco mosaic virus* and *Cucumber green mottle mosaic virus* antigens in arctic moss. This was the first proof that nonvascular plants could host herbaceous viruses. Furthermore, constructs containing viral sequences have been shown able to express and replicate in *Chlamydomonas reinhardtii* algae cells, thus demonstrating the compatibility of these genes with the algal expression/replication system [16], [17]. Recently, two plant viruses were detected in several lichens and in their algal *Trebouxia* sp. photobionts: an Apple mosaic virus (genus Ilarvirus) and another virus related to Ivy latent virus (putative Cytorhabdovirus) [18]. Based on these data, we cannot exclude higher plant viruses from the list of possible algae pathogens.

In addition to viruses joined with water-living organisms, every virus released from dead organisms could in fact subsequently reach surface fresh water and marine environments [19], [20]. In a metagenomic analysis of viruses in reclaimed water sequences of novel DNA bacteriophages, eukaryotic viruses similar to plant single-stranded DNA Geminiviruses and Nanoviruses as well as RNA viruses related to the families *Comoviridae*, *Potyviridae*, *Sequiviridae*, *Tombusviridae*, and *Reoviridae* and the genus *Tobamovirus* were found [21]. This implies that water may play a role in the dissemination of at least highly stable viruses.

*Cauliflower mosaic virus* (CaMV) was the first plant virus to be discovered to contain DNA as genetic material and the first virus to be sequenced completely [22]. It is disseminated worldwide in temperate regions and is transmitted by several aphid species. Transmission by other vector type or by pollen has never been reported in nature, but CaMV can be readily transmitted mechanically to a host plant [23]. Members of the *Brassicaceae* have been reported as systemic hosts, but B29, W260, Japan-S, and NY8153 CaMV isolates are able to infect also *Solanaceae* species *Nicotiana clevelandii* and *Datura stramonium* [24], [25]. CaMV probably spread from a single population around 400–500 years ago and is known in four geographically distributed lineages [26].

In this paper, we first demonstrate the presence and multiplication of CaMV in single-cell *Trebouxia* algae and then prove that algae could be a natural host for this herbaceous plant virus.

## Material

*Trebouxia aggregata* (strain 219–1d) isolated from *Xanthoria parietina* was obtained from the Culture Collection of Algae at Göttingen University, Germany (SAG collection). Algae *Chlorella vulgaris* Beijerinck was from the Culture Collection of Autotrophic Organisms (CCALA, ref. No. 902), Institute of Botany, Třeboň, Czech Republic. Data on the other experimental strains are summarized in Table 1.

**Table 1:** List of algal strains used in this work.

CCALA ref. No.	name	order	location	habitat	CaMV presence/AC No.:
260	*Chlorella sorokiniana*	Chlorellales	Slovakia	thermal spring	−
266	*Chlorella vulgaris*	Chlorellales	Czech Republic	irrigation canal	−
788	*Chlorella vulgaris*	Chlorellales	Netherlands	eutrophic pond	KM502556
902	*Chlorella vulgaris*	Chlorellales	Tajikistan	thermal springs	−
333	*Dictyosphaerium tetrachotomum*	Chlorellales	Slovakia	peat bog in sphagnum	−
356	*Graesiella vacuolata*	Chlorellales	USA	tree bark	KM502557KP432259
363	*Koliella sempervirens*	Chlorellales	Slovakia	fishpond	−
252	*Parachlorella kessleri*	Chlorellales	Russia	unknown	±
426	*Pseudococcomyxa simplex*	Chlorellales	Czech Republic	soil	KM502558KP342258
336	*Diplosphaera cf*. *chodatii*	Prasiolales	Czech Republic	soil, forest	KM502559
495	*Stichococcus chloranthus*	Prasiolales	Germany	unknown	KM502560
910	*Elliptochloris cf*. *subsphaerica*	Microthamniales	Svalbard	soil	KM502561
368	*Microthamnion kuetzingianum*	Microthamniales	Slovakia	peat bog	±
396	*Oonephris lacustris*	Oocystales	Czech Republic	unknown	KM502562
901	*Gloeocystis vesiculosa*	Oocystales	Italy	stone	−

− = negative, + = positive, ± = intermediate

## Methods

### Cultivation of algae

Algae were cultivated on 1.5% agar plates with 3xN (meaning three times more nitrogen content in the form of NaNO_3_) and Bold’s basal medium [27] supplemented with peptone (10 g/l) and glucose (20 g/l) with 12 h photoperiod at 20°C for 30 days.

### Biological test and virus purification

For infectivity tests, true leaves of Chinese cabbage were first mechanically inoculated with alga suspension in 0.1 M phosphate buffer, pH 7.4, and cultivated in an insect-proof glasshouse. Symptoms were evaluated 14 days after inoculation. For purification, leaves were ground in 0.5 M potassium phosphate buffer (pH 7) containing 0.75% sodium sulfite. After filtration, 2.5% Triton X100 and 1 M urea were added and stirred overnight. One cycle of differential centrifugation was used to concentrate the virus. Purification was completed by rate zonal centrifugation in 10–40% sucrose density gradient and high-speed centrifugation pelleting of the virus fraction [28], [29].

### Nucleic acid isolation and transcription

DNA and RNA was isolated from a pinhead amount of alga culture growing on agar plate using a DNA plant kit and RNA plant kit, respectively (Macherey Nagel, Germany) according to the manufacturer’s recommendation. The isolation includes 15 min on-column of enzymatic RNase and DNase treatment, respectively. DNA from lichen samples was isolated using the Wizard Magnetic 96 DNA Plant System kit (Promega, USA) from about 100 mg of dry lichen thalli in 50 μl of sterile water. The iScript cDNA synthesis kit (Bio-Rad, USA) was used for cDNA synthesis.

### Amplification

Virus screening was performed using CaMV-specific primers Ca355 5′-ACCAAATTATTGATCTAACC-3′ and Ca356, 5′-AAGATAGTCTTCTCTATTGG-3′ from the CaMV capsid protein gene (nucleotide position 2318–2739 on the D/H isolate). PCR products of expected size were gel-purified and sequenced with primers used for amplification by the BigDye Terminator v3.1 Cycle Sequencing kit (Life Technologies, USA). The complete CaMV genome sequence was obtained by amplification, cloning, and sequencing.

Presence of viral transcript in algae was performed using primers Ca339 5′-AGGACCTAACAGAACTCGCCG-3′ and Ca335, 5′-TAGAGGAAGGGTCTTGCGAAGG-3′ from the 35S promoter region (nucleotide position 6910–7389) and Ca439 5′- CAGCCAAAGGTAATCTCGCA-3′ and Ca471 5′- CATTGTTTCCTATTTGAAGACTATTACC-3′ from the movement protein gene (nucleotide position 864–1256).

### Alignment and sequence analysis

Nucleotide sequences and their *in silico* transcribed amino acid sequences were compared using blastn and blastp with GenBank data. Recombination analysis in the genomic sequences was performed using programs implemented in RDP4 [30]. MEGA5 [31] and SplitsTree 4 [32] were used for phylogenetic analysis and tree construction.

### Cocultivation with virus

Purified CaMV (about 10 μg/ml) was applied to CaMV-free *Chlorella vulgaris* (CCALA ref. No: 902), and *Pseudococcomyxa simplex* (CCALA ref. No. 426) growing on plates. It was spread evenly onto each plate using a sterile spatula, then cultivated overnight with a 12-h photoperiod at 20°C and replanted 5 times over a 2-week period. Six month after application of CaMV, pinhead amount of alga culture was resuspended in Bold’s basal medium, incubated 8 hours with 1:1000 dilution of CaMV antibody (Loewe Biochemica, Germany) at 10°C and replanted on agar plates. DNA was isolated as above 10 days later and PCR test with Ca339/Ca335 and Ca439/Ca471 primers was performed.

### Electron microscopy and gold labeling

Thin sections were prepared from plate-growing algal cells. The sections were placed on nickel grids, probed 1 hour with rabbit CaMV antibody (Bioreba AG, Reinach, Switzerland) in dilution 1:100, rinsed three times and incubated 1 hour with a gold-conjugated anti-rabbit IgG (10 nm) (Aurion, Wageningen, the Netherlands) diluted 1:40 in incubation buffer as recommended by the supplier [33]. After rinses, the grids were stained with 0.5% uranyl acetate and observed in a JEOL JEM-1010 electron microscope.

### Photochemistry

The culture infected with CaMV 219–1d and the noninfected controls of *Chlorella vulgaris* were prepared in five replicates. The cultures were all inoculated on the same day in order to exclude changes resulting from different ages of cultures. The measurements of photochemical activity as a proxy for viability were performed using a FluorCam MF-800 fluorescence-imaging camera (Photon Systems Instruments, Czech Republic) in accordance with [34]. Irradiance was measured using a LI-250A light meter equipped with a LI-190 quantum sensor (LI-COR Biosciences, USA). A protocol for maximum quantum yield (F_V_/F_M_) measurement was applied. Before measurements, the samples were adapted to darkness for 15 min. Red measurement pulses were adjusted according to culture chlorophyll content to obtain an optimal signal-to-noise ratio. Measurement pulses lasted 10, 20, or 33.33 μs with irradiance of 0.89, 1.81, and 3.02 μmol m^−2^ s^−1^, respectively. Measurement of minimal fluorescence (F_0_) was for 1.75 s, followed by the application of a strong saturation pulse of blue light with duration of 960 ms and irradiance of 4500 μmol m^−2^ s^−1^ in order to obtain maximal fluorescence (F_M_). F_V_/F_M_ was calculated using FluorCam 7 software (Photon Systems Instruments, Czech Republic) according to the following equation [35], [36]:

$${\text{F}}_{\text{V}}{\text{/F}}_{\text{M}}\text{= }({\text{F}}_{\text{M}}-{\text{ F}}_{\text{0}}){\text{/F}}_{\text{M}}\text{,}$$

where F_0_ is minimal fluorescence in darkness and F_M_ is maximal fluorescence after the saturation pulse. F_V_/F_M_ values were averaged for the entire culture area.

Statistical analyses were performed using Statistica 10 software (StatSoft, USA). The null hypothesis stated that there are no differences in F_V_/F_M_ among the infected cultures and controls. Before data processing, outlying values were excluded from the evaluation using K-criterion for outlying observation with *p* = 0.05. One-way ANOVA was used to analyze differences. Results were considered significant if their *p*-value was lower than 0.05.

## Results

The *Trebouxia aggregata* alga, which is a photosynthesis partner in many lichen species, had been isolated by A. Quispel [37] from *Xanthoria parietina* lichen prior to 1943 and maintained as axenic culture no. 219–1d in the SAG collection (http://sagdb.uni-goettingen.de/detailedList.php?str_number=219-1d). Two different submissions of this culture obtained within 1 year were repeatedly positive for CaMV in PCR with different primer combinations.

**Fig 1:**
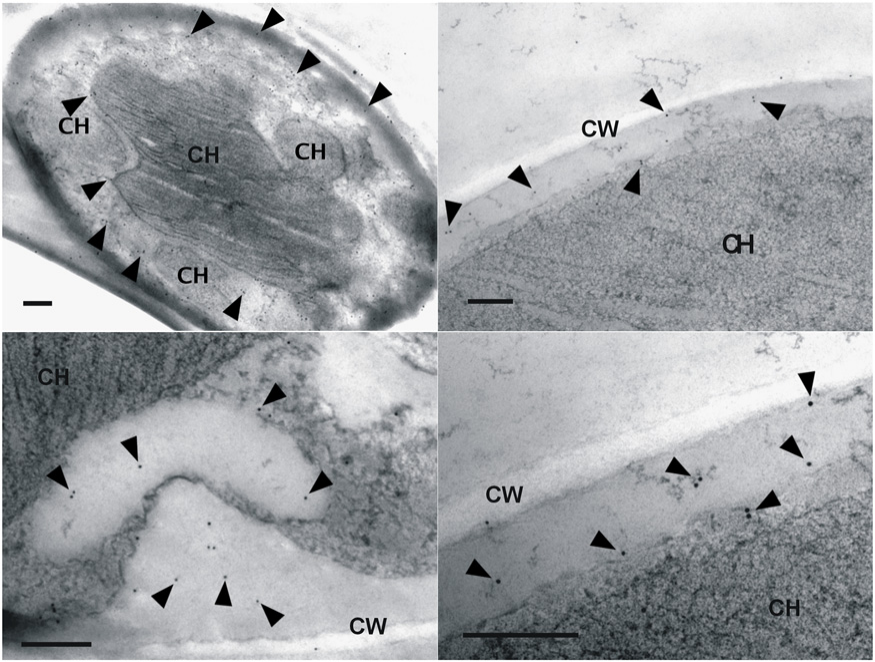
Immunodetection of CaMV with colloidal gold in *Trebouxia aggregata* alga. Thin sections were prepared from plate-growing algal cells, treated with rabbit anti-CaMV antibody, then with a gold-conjugated anti-rabbit IgG, contrasted with uranyl acetate and examined with a Jeol JEM-1010 electron microscope. 10 nm particles were detected in cytoplasma adjacent to cell, and very rarely in central chloroplast. Bars = 500 nm, CW = cell wall, CH = chloroplast.

In CaMV immunogold labeling, the gold particles were detected in cytoplasma and very rarely in chloroplast, which in mature cells assumed central position with lobes spreading to the cell periphery (Fig. 1). Occurrence of multiple signals in cytoplasma means not only presence of the virus shortly after infection, but also presence after expected reverse transcription, generation of viral particles and spread in this cell compartment. CaMV-specific products were amplified from viral cDNA from *T*. *aggregata* and free-living *Graesiella vacuolata* and *Pseudococcomyxa simplex* algae with Ca339—Ca335 and/or Ca439—Ca471 primers. The sequence from *T*. *aggregata* was 100% identical with complete genome sequence of this isolate (GenBank AC No.: KF550287), that of *G*. *vacuolata* (AC: KP342259) and *P*. *simplex* (AC: KP342258) were 97 and 98.1% identical with the sequence of CaMV D/H isolate, respectively.

Alga growing on the agar plate were scraped off, resuspended in 0.1 M phosphate buffer (pH 7.0), then mechanically inoculated onto Chinese cabbage leaves. CaMV CB1 isolate from cauliflower [38] and mock inoculation with buffer were used in parallel. Two weeks after infection, the CB1 isolate produced light green mosaic symptoms. Plants inoculated with 219–1d showed leaf wilting and rolling.

### Sequence analysis

The complete genome of the 219–1d isolate (AC: KF550287) is 8020 nt long and contains 7 major ORFs corresponding in size and position to known isolates. ORF I–V are contiguous, but ORF VI and VII are not. The large intergenic region between ORF VI and VII contains the pregenomic 35S promoter, RNA polyadenylation signal, and several transcriptional GTGG^A^/_T_ enhancer signals [39]. The small intergenic region between ORF V and VI contains the 19S promoter. In the best alignment, the complete genome of 219–1d isolate differs by 25 nt (99.7% identity) from the CB1 isolate, by 173 nt (97.8% identity) from the D/H isolate, and by 204 nt from the CRO180A isolate (97.5% identity). Seven (RDP, GENECONV, BOOTSCAN, MAXCHI, CHIMAERA, SISCAN, and 3SEQ) of nine recombination detection programs detected that 219–1d and CB1 are recombinants with sequence AB863172 as putative parental isolate. The recombination sites were detected inside ORF VI in the range of nt positions 6040–7476. Recombination has been a common feature of CaMV evolution [40], and the closest isolates D/H, CRO180A, and TUR59 also are recombinants in this gene [26]. Outside the CRO180A which was from oilseed rape (*Brassica napus*) the remaining closest isolates were from cauliflower (*B*. *oleracea* var. *botrytis*) and CB1 only is recorded to induce local symptoms on *Nicotiana clevelandii* [38].

**Fig 2:**
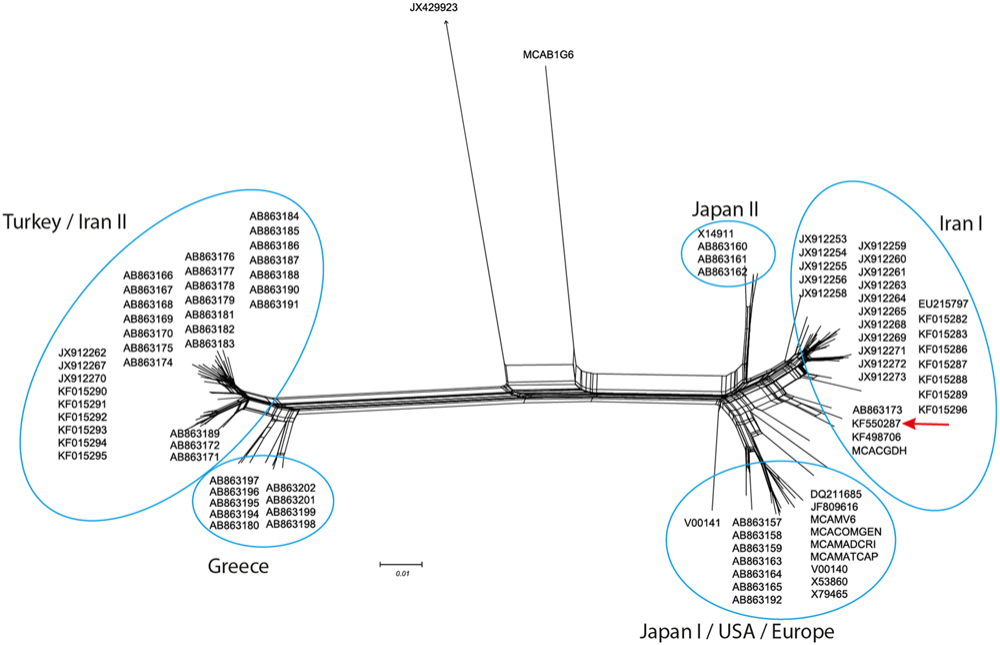
Neighbor-Net network analysis among CaMV isolates based on ORF VI and performed using SplitsTree4. Formation of a reticular network is suggestive of recombination. Horseradish latent virus (JX429923) is used as the outgroup. Position of 219–1d isolate in Iran I cluster is marked.

Formation of a reticular network after the Neighbor-Net network analysis of ORF VI performed using SplitsTree4 is suggestive of recombination. The 219–1d isolate is clustered in the Iran I subgroup together with the TUR59 and D/H isolates that are its closest relatives (Fig. 2).

SLAC, FEL, and MEME tests for positive/negative selection at individual codons of ORF IV and ORF VI (web server www.datamonkey.org) revealed no site or sites to be positively selected or under episodic diversifying selection and specific for the alga isolate. In sequence analysis based on ORF VI, the known *Nicotiana*-infecting isolates are placed in different clusters: the Japan-S isolate in Japan II cluster, while B29, W260, and NY8513 in JapanI/USA/Europe cluster [26]. There is no position specific for these isolates and alongside lacking in isolates classified together. We therefore concluded that the algal environment had not driven the fixing of any specific mutation in the 219–1d isolate.

### Photochemistry/viability

**Fig 3:**
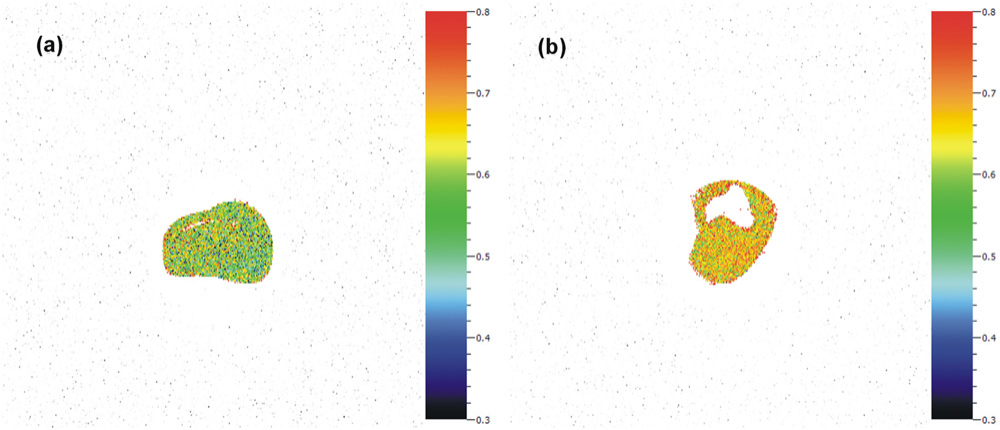
Viability comparison of CaMV-infected and noninfected algae cultures. Heterogeneity of F_V_/F_M_ in experimental infected a) and control b) cultures. False color scale—black/blue correspond to the lowest values and orange/red to the highest ones. Higher values indicate a better physiological state of the culture. Resolution is 0.046 mm^2^ per pixel. Detection size is 0.21 mm.

Although F_V_/F_M_ was not homogenous in the cultures (Fig. 3), fluorescence measurements revealed that F_V_/F_M_ was 14% lower for infected cultures (; 0.576 ± 0.061 in infected and 0.670 ± 0.038 in control cultures; one-way ANOVA, *F* = 6.77, *p* = 0.032). The decrease in F_V_/F_M_ may indicate slight stress in the infected culture caused by infection since there were no other differences in cultivation conditions.

### CaMV in different algae

Other algae species and strains from the Chlorellales, Prasiolales, Microthamniales, and Oocystales were PCR tested with primers Ca355 and Ca356 for the presence of CaMV. Strains with different virus content as well as virus-free strains were detected in each taxonomic group (Table 1). Amplified products from strains with high virus content were sequenced and BLAST search confirmed the CaMV identity of the obtained sequences. The virus was detected in CaMV-free Chlorella and Pseudomonas algae treated with CaMV after five passages and after antibody treatment to remove surface CaMV.

## Discussion

The algae photobiont had been isolated from a strain of the globally distributed lichen species *Xanthoria parietina* and maintained in a collection as an axenic strain for more than 70 years. No fungus growth was visible when the culture was cultivated on 3xN CBB agar, and the lichen fungus can therefore be excluded as the source of the virus. We assume that the last opportunity for the virus to enter algal cells was before preparation of the algal culture. Possible laboratory contamination was excluded by testing two independent batches of the 219-1d strain obtained within 1 year’s time (both of which batches then contained CaMV) and by physical separation of areas in the laboratory where the DNA isolation and manipulation of PCR amplicons were conducted.

Many open questions remain about the cohabitation of the virus and the (lichen) alga. First, what is the origin of the virus? We analyzed the algal virus genome in detail and revealed that it is highly similar to the virus genome of the known European CaMV isolate D/H from cauliflower, including its recombination nature. Moreover, there is no sequence proof for an algal origin of the virus. All phylogenetic analyses performed independently for the 7 major ORFs of CaMV classified the 219–1d isolate close to the European CaMV D/H isolate and no positively selected codon was found in the 219–1d genome. We conclude that the virus found in the alga does not represent a new or independent evolutionary lineage. More probably, it represents an accidental infection by a current strain in this host.

Second, what is the method of acquiring CaMV? We did not study this process directly, but the entry of the virus should not be very restricted inasmuch as this work demonstrated that simple addition of purified virus to algal culture led to infection. This process could not be limited to *Trebouxia* or *Chlorella* algae, as many other algal species have been established as containing the virus. The PBCV-1 chlorovirus (family *Phycodnaviridae*) initiates infection by specific attachment to the *Chlorella variabilis* cell wall receptor with a unique spike structure protruding from the surface of the virion [41] followed by host cell degradation by a virion-associated enzymes and fusion of viral and algal membranes. Rapid depolarization of the host membrane triggered by a virus-encoded K^+^ channel results in reduction of turgor pressure, which may aid ejection of viral DNA into the host [42]. However, there is no evidence for a specific entry mechanism of CaMV, although P1 is known to interact with the cell wall-associated pectin methylesterase of tobacco [43]. No cell-degrading enzymes encoded by CaMV as well as no K^+^ channel-forming proteins facilitating the wall penetration are known.

Third, is there a reasonable chance for the lichen alga to encounter CaMV? CaMV is reported worldwide from temperate regions where its hosts grow [28]. In fact it also has been reported from arid and tropical African countries (Egypt, Sierra Leone, Tanzania, Zimbabwe—[44], Hawaii [45], Israel [46], and recently from arid regions in Iran [47]. Natural transmission and spread could occur via any one of 27 aphid species [28], but it is difficult to imagine worldwide dissemination only by an insect vector. CaMV viral particles are very stable in cauliflower sap [28], and these could disseminate as an airborne biological particulate after the host plant’s death and with the help of wind and atmospheric circularization. Airborne spread of human and animal viruses causing respiratory diseases over short distances (measurable in meters) is common and widely known. Viruses are also expected to be ubiquitous in the near-surface atmosphere and their abundance in different land types has been assessed in a range between 10^6^ and 10^7^ virus particles per cubic meter [48]. Metagenomic analyses have revealed sequences of small ssDNA and ssRNA viruses related to the families *Circoviridae*, *Inoviridae*, *Nanoviridae*, *Geminiviridae*, *Microviridae*, and *Tombusviridae* as well as large dsDNA and dsRNA viruses related to the *Polyomaviridae*, *Rhabdoviridae*, *Herpesviridae*, and *Poxviridae* [48]. Cosmopolitan like CaMV, *Xanthoria parietina* L. (common orange lichen, maritime sunburst lichen) is a foliose lichen growing on rocks or tree bark [49] and *Trebouxia* sp. algae are the most frequent photobionts associated with this fungus [50]. Most lichen algae are only facultative photobionts and occur free-living in nature as epiphytes, endoliths, or soil algae [3]. We assume that at this life stage they are accessible for virus acquisition if it is present in the close vicinity. Lichen containing virus-infected algae could then form in the process of relichenization when germinating fungal ascospores join with free infected algae. This event could be promoted by the presence of both the fungus and alga in the digestive system of oribatid mites’consumers [51].

Finally, but not of least importance, is the question of what physiological effect does the virus have on the algae? In turnip as well as in *Nicotiana* protoplasts the CaMV is slow-replicating virus and its replication kinetics are more probably independent of the host cell [52]. The amount of virus in algae is also very low as it is detectable in high (40) cycle numbers only. In this case, the infection resembles viral persistent infection. To date, however, no such infection has been detected in algae [14]. We therefore suppose that no acute infection impact occurred in the conditions under which the 219–1d strain was cultivated during the 70-year period. While it is known that viral infection decreases photosynthetic activity in infected cells [53], [54], [55], [56], fluorescence measurements in virus-infected algae are rare. Nevertheless, the intensity and response rate of photosynthetic processes to viral infection seem to be virus- and strain-specific. For example, HaRNAV infection was shown only slightly to reduce the maximum quantum yield in *Heterosigma akashiwo* while WBs1 and OIs1 infections caused a steep decline in the maximum quantum yield over the same period [49]. Our fluorescence measurements in *Ch*. *vulgaris* revealed a slight inhibition of photosynthesis in the infected cultures. Considering the inter-replicates variability range of 1–10%, the observed decrease in F_V_/F_M_ of 14% for infected cells may not play an important role in survival under optimal conditions. Under suboptimal and limiting conditions, however, the effect of viral infection may be more profound.

This research provides the first proof for the natural presence of CaMV in algae and the first demonstration of artificial infection of algae with this virus. Airborne, free-living algae should be considered an important plant virus shuttle that is in addition to dispersion of free viral particles.
